# Supplementary material for: The Role of IL-6 in Inner Ear Impairment: Evidence from 146 Recovered Patients with Omicron Infected in Tianjin, China
Source: J Clin Med. 2023 Jan 31;12(3):1114. doi: 10.3390/jcm12031114 (PMC9917594; doi:10.3390/jcm12031114)
Supplement: Supplementary file 1 [file jcm-12-01114-s001.zip › jcm-2129112-supplementary.pdf]

Supplementary

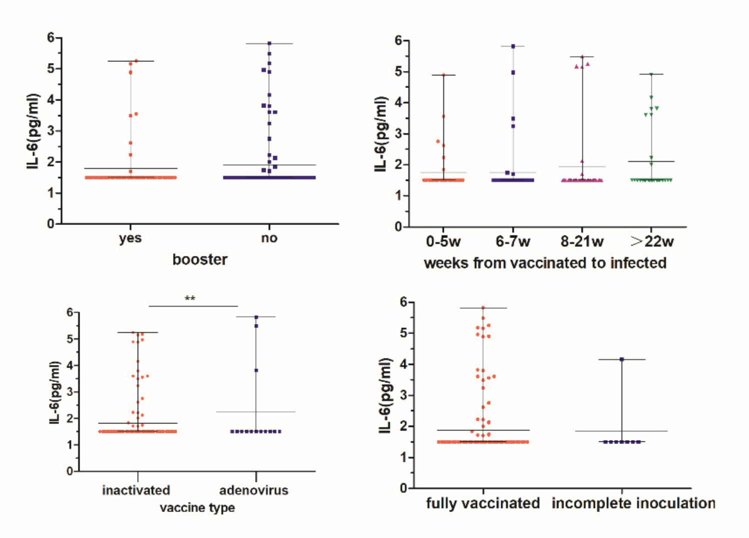

Figure S1. Comparison of IL-6 for different characteristic of vaccine .

Table S1. DPOAE results and patients' vaccination statuses.

	N	fail	pass	P
Booster vaccinated				
Yes	59 (40.4%)	13 (22.0%)	46 (78.0%)	0.364
No	87 (59.6%)	14 (16.1%)	73 (83.9%)	
Fully vaccinated				
Yes	138 (94.5%)	25 (18.1%)	113 (81.9%)	0.626
No	8 (5.5%)	2 (25.4%)	6 (75%)	
Vaccine type				
inactivated	132 (90.4%)	21 (77.8%)	111 (94.9%)	0.004
adenovirus	12 (8.2%)	6 (22.2%)	6 (5.1%)	
Weeks from vaccinated to infected				
0–5	34 (23.6%)	9 (33.3%)	25 (21.4%)	0.194
6–7	48 (33.3%)	6 (22.2%)	42 (35.9%)	
8–21	35 (24.3%)	9 (33.3%)	26 (22.2%)	
> 22	27 (18.8%)	3 (11.1%)	24 (20.5%)	

The Chi-square test was used to determine the differences in categorical data between the different groups.
